# An Indolent Ulcer, Speedily Cured by the Electric Moxa

**Published:** 1851-07

**Authors:** 


*An Indolent Ulcer, speedily cured by the Electric Moxa.*—Mr. Bransby B. Cooper, in a clinical lecture, reported in the London Medical Times, 24th May, 1851, gives an account of a rapid cure of an obstinate indolent ulcer, of two years standing, by the use of electric moxa. The mode of applying this agent is as follows: "There is no complicated apparatus required; no obstacle, no impediment to its application in the hands of the General Practitioner. All that is necessary is a piece of zinc and silver foil, each about the size of a crown piece, which, connected together with a few inches of copper wire, forms the metallic portion of the battery, which is completed by the intervention of the living tissues.

A blistered surface being made a short distance from the sore, the zinc plate placed on this, and the silver plate on the sore itself, a simple galvanic battery is at once formed, and a circuit of electricity established, passing in a direction from the zinc or generating plate by molecular decomposition through the fluids of the tissue to the silver or conducting plate, and thence reconducted to the zinc whence it originated, by (according to the modern doctrine) a polar arrangement of the constituent molecules of the copper wire, thus completing the circle. The battery is excited by the chloride of sodium contained in the animal fluids,—the chlorine being set free at the zinc plate, combines with this metal, forming chloride of zinc, which, acting as an escharotic, gives rise to the eschar that is produced; whilst the sodium, being liberated at the silver plate, to which it gives up its positive influence, immediately decomposes water, combines with its oxygen, and sets free hydrogen. It is not absolutely requisite to denude the dermis by means of a blister previous to the application of the moxa; for if the cuticle be moistened with a solution of chloride of sodium or common salt, when the zinc plate is to be applied, a galvanic current is established, and chloride of zinc formed, which soon destroys the cuticle, and produces an eschar of the subjacent parts, if its application be persisted in."

